# Exploring the association of physical activity with the plasma and urine metabolome in adolescents and young adults

**DOI:** 10.1186/s12986-023-00742-3

**Published:** 2023-04-05

**Authors:** Samuel Muli, Christian Brachem, Ute Alexy, Matthias Schmid, Kolade Oluwagbemigun, Ute Nöthlings

**Affiliations:** 1 Nutritional Epidemiology, Department of Nutrition and Food Sciences, University of Bonn, Friedrich-Hirzebruch- Allee 7, 53115 Bonn, Germany; 2 Institute for Medical Biometry, Informatics and Epidemiology (IMBIE), University Hospital Bonn, Venusberg Campus 1, 53127 Bonn, Germany

**Keywords:** Metabolomics, Physical activity, Adolescents, Young adults

## Abstract

**Background:**

Regular physical activity elicits many health benefits. However, the underlying molecular mechanisms through which physical activity influences overall health are less understood. Untargeted metabolomics enables system-wide mapping of molecular perturbations which may lend insights into physiological responses to regular physical activity. In this study, we investigated the associations of habitual physical activity with plasma and urine metabolome in adolescents and young adults.

**Methods:**

This cross-sectional study included participants from the DONALD (DOrtmund Nutritional and Anthropometric Longitudinally Designed) study with plasma samples *n* = 365 (median age: 18.4 (18.1, 25.0) years, 58% females) and 24 h urine samples *n* = 215 (median age: 18.1 (17.1, 18.2) years, 51% females). Habitual physical activity was assessed using a validated Adolescent Physical Activity Recall Questionnaire. Plasma and urine metabolite concentrations were determined using ultra-high-performance liquid chromatography-tandem mass spectroscopy (UPLC-MS/MS) methods. In a sex-stratified analysis, we conducted principal component analysis (PCA) to reduce the dimensionality of metabolite data and to create metabolite patterns. Multivariable linear regression models were then applied to assess the associations between self-reported physical activity (metabolic equivalent of task (MET)-hours per week) with single metabolites and metabolite patterns, adjusted for potential confounders and controlling the false discovery rate (FDR) at 5% for each set of regressions.

**Results:**

Habitual physical activity was positively associated with the “lipid, amino acids and xenometabolite” pattern in the plasma samples of male participants only (β = 1.02; 95% CI: 1.01, 1.04, *p* = 0.001, adjusted *p* = 0.042). In both sexes, no association of physical activity with single metabolites in plasma and urine and metabolite patterns in urine was found (all adjusted *p* > 0.05).

**Conclusions:**

Our explorative study suggests that habitual physical activity is associated with alterations of a group of metabolites reflected in the plasma metabolite pattern in males. These perturbations may lend insights into some of underlying mechanisms that modulate effects of physical activity.

**Supplementary Information:**

The online version contains supplementary material available at 10.1186/s12986-023-00742-3.

## Introduction

A large body of evidence emphasizes health benefits of physical activity and an active lifestyle, from early childhood through adolescence into adulthood. High level of physical activity has been associated with lower risk of cardiovascular diseases [1, 2], type 2 diabetes and metabolic syndrome [3, 4], all-cause mortality [1, 2, 5] and mental disorders [6]. The effect of physical activity on the immediate fuel utilization through aerobic and anaerobic metabolism is well elucidated [7]. However, molecular mechanisms modulated by physical activity, or through which regular physical activity benefits overall health is less clear.

Physical activity is heterogeneous behavior, varying in type of exercise, frequency, intensity, and duration. Some studies have reported marked individual variability in the responsiveness to exercise training [8, 9], suggesting idiosyncratic biological responses and physiological gains. In one study, researchers identified a set of plasma proteomic profiles whose baseline composite score predicted an individual’s ‘trainability’ and how they benefit or respond to an exercise training protocol [10]. Genetic factors may be important even though their mediating role is not firmly established [11–13]. Previous studies have also identified potential quantitative trait loci linked to an individual’s response to exercise training such as changes in plasma insulin [14], glucose and insulin metabolism [15], and lipid and lipoprotein levels [16].

As research in exercise physiology continues to expand, metabolomics has become a popular technique for mapping molecular responses to physical activity and exercise-associated metabolism. For example, global shifts in the levels of lipids and lipid-related metabolites during and after exercise training has been described in several metabolomics-based studies [17–19]. There is also circumstantial evidence linking physical activity with amino acid metabolism in the skeletal muscles, where higher levels of habitual leisure time physical activity were associated with an elevated branched-chain amino acids (BCAAs) catabolic processes [20] and reduced concentration of circulating BCAAs in serum [21]. Given that an elevated concentration of circulating BCAAs is associated with obesity and metabolic syndrome [22–25], these studies underscore the utility of metabolomics in exploring physiological responses to physical activity and the biochemical pathways to health status.

There is evidence of clear and concrete benefits for encouraging development and tracking of leisure time physical activity over the life course [26]. But there are far fewer metabolomics-based studies focused on physical activity in adolescents and young adults. Two small intervention studies examined short-term exercise-induced physiologic effects on the metabolome of young adults [27, 28] while one study explored longitudinal associations of physical activity and serum circulating amino acids concentration in peripubertal girls [29]. In the present study, we explore potential associations of habitual leisure time physical activity (LTPA) with the plasma and urine metabolome in adolescents and young adults.

## Methods

### Study design

This analysis involved participants from the Dortmund Nutritional and Anthropometric Longitudinally Designed (DONALD) study. Detailed description of the study design and population is provided elsewhere [30]. Briefly, the DONALD study is an open-cohort study that was started in 1985, with the primary objective of assessing diet and nutrition and their complex interrelations with metabolism, growth and development from infancy to early adulthood. Regular annual assessments include dietary intake using 3-day weighed dietary records (3d-WDR), anthropometric and medical measurements, 24-h urine sample collections, and interviews on lifestyle factors such as physical activity. The first examination starts at the age of three months, with three assessments planned during the first year, two annual assessments in the second year, and afterwards, one annual assessment until the age of eighteen, after which, examinations are performed once in a five-year period. For this analysis, unless otherwise specified, we used participants’ data before or at the same follow-up visits as the blood and urine sample collection.

### Study participants

The present cross-sectional analysis included participants from singleton, full term births (36–42 weeks of gestations) and had a birthweight of at least 2500 g. We used two subsets of the DONALD study participants based on the untargeted metabolome profiling of either the blood (plasma) samples (*n* = 418) or the urine samples (*n* = 369), described in previous studies [31, 32]. Overall, a sample of *n* = 365 for plasma and *n* = 215 for urine who had physical activity measurements were included. Out of these, *n* = 136 had both plasma and urine measurements.

### Assessment of physical activity

Habitual LTPA as assessed in the DONALD study reflects physical activities performed at the discretion of the participant that are not essentially part of their daily living. These included sporting and recreational activities in their leisure time as individuals, with friends, in organized groups or sports clubs and any other after-school-but-in-school physical activity, but excludes activities during physical education. Using a questionnaire based on the validated Adolescent Physical Activity Recall Questionnaire (APARQ) [33], participants were required to estimate, on average, the amount of time they spent per week in organized and unorganized sports within the last 12 months. From the age of 12 onwards, participants were interviewed in person or with the help of their parents if accompanied, to find the most possible correct responses. The amount of total reported LTPA was calculated as weekly energy expenditure given by metabolic equivalent of task (MET)-hours per week. After exploratory data analysis, we used the Tukey’s interquartile range rule for outlier detection and excluded the extreme physical activity observations. For this analysis, we used LTPA assessment closest in time before blood and urine collection, as the exposure variable. The time difference (in days) between LTPA assessment and urine and blood draw collection dates was also determined and considered in data analyses.

### Metabolite measurement

Untargeted metabolomics analyses of plasma samples (*n* = 418) and 24-h urine samples (*n* = 369) were performed by Metabolon Inc. (Morrisville, NC, USA) using Ultra-high-performance liquid chromatography-tandem mass spectroscopy (UPLC-MS/MS) methods. Briefly, through Metabolon’s global metabolomics platform and a host of standardized processes related to sample accessioning, sample preparation, instrumental analysis, peak quantification and batch correction, metabolites are characterized in reference to their database of over 3300 registered chemical compounds and mass spectral entries of structurally unnamed biochemicals. In the plasma, 1042 features were annotated, of which 811 compounds were of known chemical identity and 231 of unknown structural identity. 1407 biochemical compounds were annotated in the urine of which 940 compounds were of known or named biochemical identity and 467 compounds were of unknown structural identity. For a detailed description of these methods and analytic quality control procedures, we refer the interested reader to Additional file **1** in supplementary materials.

### Assessment of other variables

Demographic data as well as socioeconomic and lifestyle factors were collected at study entry and during annual measurements. These included sex, age, dietary intake using 3-day weighed dietary records (3d-WDR), body mass index (BMI, calculated as participant’s weight divided by the square of height in meters – Kg/m^2^). From the dietary intake, we calculated total daily energy intake (kilocalories/day) and macronutrients (carbohydrates, fat and protein) following our in-house food composition database [34]. Other covariates assessed include smoking and alcohol status, household factors (i.e., smoking household – yes/no) and social economic factors (i.e., maternal occupation and educational level). These covariates are potential confounders and were included a priori based on existing literature [35–37].

### Statistical analyses

Participants’ characteristics were summarized using median with 25% and 75% percentile for continuous variables and count (percentage) for categorical variables. All data processing and downstream analyses were sex-stratified because of the well-established sex differences in physical activity levels and intensity [2, 38, 39], human metabolome [40–42] and the interrelationship between some metabolites such as tryptophan [43]. As a data processing step, we excluded metabolites with more than 20% missing values as per the “80% rule” described in [44]. This threshold strikes a good balance between filtering out features and preserving “data quality” for which imputation should work reasonably well [44, 45]. In plasma samples, this exclusion represented 251 and 265 metabolites in males and females, respectively and in urine samples, this represented 222 and 229 metabolites in males and females, respectively. Most metabolites were not normally distributed; thus, concentrations were natural-log transformed and standardized to a mean of zero [45]. All missing data were imputed through a Random Forest (RF) algorithm using *missForest* R package. The RF-based imputation has been show to outperform other imputation methods for metabolomics data, especially, when the patterns of missingness are unknown [46].

We used principal component analysis (PCA) to reduce the dimensionality of the metabolites data, transforming them linearly to a smaller set of composite factors (i.e., principal components - PCs) that are orthogonal and uncorrelated while still explaining most of the variance in the original data [47]. The function “prcomp ()” from the *R Stats* package was used to perform PCA of the covariance matrix. The PCs that explained a cumulative variance of at least 70% of total variability were retained [47]. These PCs represented metabolite patterns and were used in multivariable linear regression models.

In regression modeling of metabolome data, individual metabolites are usually analyzed one by one with correction for multiple testing. Despite the benefit of this approach in correcting for type I error rate and providing a measure of association for each metabolite, single metabolite associations might be too small to detect, and relationships among metabolites are ignored. In contrast, the PCA approach addresses these issues by incorporating most of the information of the metabolite matrix into few uncorrelated variables (PCs). As such, we were also interested in patterns of variation in the whole metabolite matrix that may be related to physical activity rather than focusing only on individual metabolites. We considered both approaches and analyzed both individual metabolites and PCA factor scores as outcome variables in multivariable linear regression models.

Thus, using multivariable linear regression, we regressed (*i*) each of the single metabolites and (*ii*) each of the PCA factor scores on LTPA scores, adjusting for other covariates. Given the high number of metabolites and the exploratory approach of these analyses, we corrected for multiple comparisons by controlling the false discovery rate at 5% for each set of regressions using the Benjamini-Hochberg procedure. For interpretation, log-transformed regression coefficients were back-transformed. All statistical analyses were performed using the R statistical software version 4.1.3.

## Results

### Description of study population

Table 1 provides a summary of the basic characteristics of the study participants. The plasma sample included 365 participants (58% females) with a median age of 18 years, BMI of 22 kg/m^2^, LTPA of 26 MET-hours/ week and energy intake of 2034 Kcal/day. The urine sample included 215 participants (51% females) with a median age of 18 years, BMI of 22 kg/m^2^, LTPA of 33 MET-hours /week and energy intake of 2135 Kcal/day.

**Table 1:** Basic characteristics of the study population

	Plasma Metabolome		Urine Metabolome	
Characteristic	**N = 365**		** N = 215**	
Sex, females^*a*^	365	213 (58.4)	215	109 (50.7)
Age at sample collection, years^*b*^	365	18.4 (18.1, 25.0)	215	18.1 (17.1,18.2)
BMI at sample collection (kg/m^2^)^*b*^	365	22.3 (20.5, 24.7)	215	21.9 (19.9, 24.2)
Physical activity (MET-h /w)^*b*^	365	26.0 (12.0, 47.0)	215	33.0 (13.0, 53.0)
Energy intake (Kcal/day)^*b*^	276	2034 (1637, 2457)	214	2135 (1759, 2518)
Protein intake (E%)^*b*^	276	13.9 (12.3, 15.7)	214	13.7 (12.2, 15.4)
Fat intake (E%)^*b*^	276	33.1 (29.4, 38.1)	214	33.0 (30.1, 38.0)
Carbohydrate intake (E%)^*b*^	276	50.0 (45.2, 55.1)	214	50.0 (45.0, 55.1)
Smoking status^a^	301		154	
Never		194 (64.5)		109 (70.7)
Former		51 (16.9)		21 (13.6)
Current		56 (18.6)		24 (15.5)
Alcohol consumption^*a*^	325		170	
Never		26 (8.0)		21 (12.3)
Former		42 (12.9)		30 (17.6)
Current		257 (79.1)		119 (70.0)
Smoking household: Yes^*a*^	260	83 (31.9)	157	49 (31.2)
Maternal occupation (working full or part-time): Yes^*a*^	283	187 (66.1)	174	138 (79.3)
Maternal education ≥ 12 years of education: Yes^*a*^	284	158 (55.6)	174	113 (52.6)

Data presented as ^*a*^ n (%) where n = count, % = percentage, ^*b*^ Median (25%, 75% percentile), BMI, body mass index calculated as weight (kg)/height (m^2^), E% indicates percentage of total energy intake per day, Physical activity reflects the leisure time physical activities in MET-h /w, metabolic equivalent of task-hours per week. Differences in N due to missing data for that variable

### Summarizing metabolites into groups using PCA

Table 2 summarizes the results of the PCA of the metabolite datasets in which the high number of plasma and urine metabolites were reduced to fewer PCs (henceforth referred to as metabolite patterns) representing patterns of correlated metabolites that may have biologically related information.

**Table 2:** Summary of plasma and urine metabolome PCA results

	Plasma metabolome			Urine metabolome		
	Metabolites	PCs	Cumulative variance (%)	Metabolites	PCs	Cumulative variance (%)
Female	777	37	70.4	1185	25	70.6
Male	791	34	70.7	1178	24	70.4

PCs – Principal components through PCA on covariance matrix. The PCs column represents the total number of principal components explaining at least 70% of variance of the metabolite data

### Associations between leisure time physical activity and metabolome

In multivariable linear regression models of single metabolites as response variables and LTPA and covariates as predictor variables, we found no associations between LTPA and the metabolites after controlling the false discovery rate at 5% in both plasma and urine samples for both sexes (all fdr-adjusted *p* > 0.05). In multivariable linear regression with metabolite patterns as response variables and LTPA and covariates as predictor variables, LTPA was positively associated with PC15 in male plasma samples (β = 1.02; 95% CI: 1.01, 1.04, fdr-adjusted *p* = 0.042) (Table 3). No other LTPA-metabolite patterns associations were found in female plasma samples as well as in urine samples for both sexes (*p* > 0.05 after correction for multiple testing). Complete results of these analyses are provided in the Additional file **2**: Table **S1**.

**Table 3:** Regression coefficients for the association between plasma PCA factors and LTPA scores in male participants

Metabolite patterns (PC factors)	Variance (%)	Cumulative variance (%)	Estimate	95% Confidence interval			
			**Β**	**Lower**	**Upper**	***p*** **value§**	***p*** **(fdr)**
PC1	7.65	7.65	0.996	0.974	1.020	0.756	0.914
PC2	6.77	14.42	0.987	0.961	1.014	0.347	0.655
PC3	5.00	19.42	0.998	0.976	1.021	0.852	0.918
PC4	4.63	24.05	1.004	0.985	1.024	0.689	0.914
PC5	3.62	27.68	0.978	0.960	0.997	**0.023**	0.391
PC6	3.10	30.78	1.011	0.993	1.029	0.241	0.556
PC7	2.82	33.60	0.999	0.983	1.016	0.918	0.918
PC8	2.68	36.28	0.988	0.972	1.005	0.156	0.556
PC9	2.49	38.77	1.013	0.997	1.029	0.122	0.556
PC10	2.20	40.97	1.003	0.988	1.018	0.714	0.914
PC11	2.03	43.00	0.992	0.979	1.006	0.250	0.556
PC12	1.92	44.92	0.992	0.979	1.006	0.261	0.556
PC13	1.82	46.74	1.009	0.995	1.023	0.197	0.556
PC14	1.63	48.37	1.003	0.990	1.017	0.627	0.914
PC15	1.58	49.94	1.022	1.009	1.035	**0.001**	**0.042**
PC16	1.55	51.49	1.003	0.991	1.016	0.595	0.914
PC17	1.50	52.99	0.989	0.976	1.002	0.088	0.556
PC18	1.39	54.38	0.997	0.984	1.009	0.584	0.914
PC19	1.36	55.74	1.011	0.999	1.024	0.066	0.556
PC20	1.29	57.03	1.010	0.998	1.021	0.106	0.556
PC21	1.18	58.20	0.999	0.988	1.011	0.895	0.918
PC22	1.15	59.36	0.999	0.988	1.010	0.866	0.918
PC23	1.12	60.48	0.994	0.983	1.005	0.258	0.556
PC24	1.08	61.56	1.001	0.991	1.012	0.807	0.914
PC25	1.06	62.61	1.003	0.992	1.013	0.596	0.914
PC26	1.04	63.66	0.994	0.984	1.004	0.225	0.556
PC27	0.97	64.63	0.994	0.984	1.004	0.232	0.556
PC28	0.95	65.58	0.998	0.988	1.008	0.751	0.914
PC29	0.91	66.49	0.999	0.989	1.009	0.801	0.914
PC30	0.90	67.39	1.005	0.995	1.015	0.322	0.643
PC31	0.86	68.25	1.001	0.992	1.011	0.782	0.914
PC32	0.85	69.10	0.994	0.984	1.004	0.211	0.556
PC33	0.81	69.91	0.996	0.987	1.006	0.411	0.736
PC34	0.78	70.69	1.005	0.996	1.015	0.262	0.556

Estimates from multivariable linear regression with principal component analysis scores (PCA factors) modelled as outcome variable and leisure time physical activity as explanatory variable. The models were adjusted for all covariates and estimates back-transformed for interpretation. **§** represents the uncorrected p-value, *p*(fdr), p value corrected for multiple comparison using the FDR method

### Metabolite patterns associated with leisure time physical activity

To describe the metabolite pattern, PC15 (Table 3), we investigated metabolite loadings and the biochemical identities of the top ranking metabolites. Note that PC15 is defined by a weighted combination of the log-transformed and centered metabolite values. Thus, the weights or loadings of the individual metabolites would be equal in absolute value if all metabolites contributed equally to PC15. We determined the median of individual metabolite contributions to PC15 and considered those above the median to represent the most significant metabolites contributing to the observed pattern. Additionally, using the *factoextra* R package, we graphically inspected individual metabolite contributions or weights. Based on this selection criteria, *n* = 82 metabolites were selected (more details on this methods provided in Additional file **3**: Figure **S1**, Figure **S2**). Notably, PC15 variation was primarily driven by metabolites from the lipid, amino acid, and xenobiotic super pathways i.e., lipids (*n* = 25) amino acids (*n* = 15) xenometabolites (*n* = 13) cofactors and vitamins (*n* = 3), carbohydrate (*n* = 2), nucleotide (*n* = 2), peptide (*n* = 1) and unknown or non-annotated metabolites (*n* = 21). We named this PC15 pattern “lipid, amino acid, and xenometabolite pattern”. Detailed information on the biochemical identities, super-and-sub pathways of the metabolites is summarized in Additional file **3**: Table **S2.**

## Discussion

This study explored sex-stratified associations between physical activity and single metabolites as well as metabolite patterns in plasma and urine samples from young adults. The major findings from our analyses was that independent of the covariates, physical activity was positively associated with the “lipid, amino acids and xenometabolite” pattern, specifically, in the plasma samples of males.

The unsupervised PCA approach created uncorrelated patterns, with each PC representing an independent pattern of variation, and metabolite weights reflecting their contribution to that pattern [47]. These patterns represent a mixture of biological processes and pathways and may reflect metabolome perturbations as a response to a physiological state, or related to a phenotype of interest [48, 49]. These may include diet, body composition, aging, physical activity among others. We emphasize the exploratory nature of our analyses; therefore, several biological explanations exist for these findings. For example, that physical activity was associated with a pattern of metabolites and not with single metabolite could reflect the complexity of the multiple biological processes involved. It could also mean that leisure time physical activity elicits only small effects on individual metabolites that are not detectable after multivariable adjustments and multiple testing corrections as observed in our single metabolite models. Therefore, we do not attribute the effects of habitual physical activity on metabolome on specific metabolites, but rather on groups of metabolites given that the observed association reflect the variation or combined effects of small changes in multiple metabolites in the pattern.

In the present study, twenty-five of the top loading metabolites in the metabolite pattern associated with physical activity are from lipid metabolism super pathway. The most represented sub-pathways were fatty acid metabolism (dicarboxylate (4), monohydroxy (2), dihydroxy (1), acyl choline (1), Acyl carnitine (1) and long-chain polyunsaturated fatty acids (1), BCAA metabolism (2), endocannabinoid (1)), bile acid metabolism (5), steroids (5), and phospholipid metabolism (2). Broadly, exercise-induced perturbations of multiple lipid-related metabolites is reported in other epidemiologic studies. Three separate experimental studies investigating exercise-induced shifts in lipids reported significant changes in the concentration of fatty acid oxidation metabolites, particularly, dicarboxylate fatty acids, monohydroxy fatty acids and acylcarnitines [17], ketones, dicarboxylate fatty acids and long-chain fatty acids sub pathways [19], and two-fold or higher increase in lipid/carnitine metabolites with significant decrease in lysolipid and bile acid metabolites [18].

In other plasma-based metabolome studies, long-term leisure time physical activity was also associated with alterations in lipid profiles, particularly, elevated concentration of high-density lipoprotein (HDL) cholesterol [21, 50], and similarly, in a 2-day ultramarathon study [51]. In line, studies investigating cardiorespiratory fitness observed significant differences in concentrations of beneficial lipid metabolites and fatty acids among individuals with high and low fitness [21, 52, 53]. We note that during physical exercise, increased demand for energy is fulfilled through oxidation of glucose or hydrolysis of triacylglycerols into free fatty acids depending on among other factors, the prandial state and intensity and duration of the exercise [54, 55].

We found that plasma glucose and mannose — which is mechanistically a glucose-associated metabolite — also loaded in the metabolite pattern associated with physical activity. Plasma glucose is extensively documented source of energy for aerobic and anaerobic metabolism. Moderate-intensity habitual physical activity is associated with lower fasting plasma glucose levels [21, 56]. Several molecular mechanisms through which physical activity promotes glucose homeostasis have also been proposed [4, 57].

Additionally, 15 of the top ranking metabolites in the pattern associated with physical activity were from amino acid metabolism super pathway. Of note, these metabolites predominantly represented the branched-chain amino acids (BCAAs) sub-pathway (i.e., leucine, isoleucine and valine amino acids). Other important amino acid-related metabolites observed included guanidinoacetic acid (GAA) which is involved in cellular energy metabolism and believed to improve exercise performance among physically active individuals [58]. Moreover, circulating kynurenine, a tryptophan catabolite is mechanistically influenced by physical activity, with studies reporting exercise-induced kynurenine pathway modulations in both animal and human populations [59]. Of note, a wide variety of studies have reported significant associations between physical activity and alterations in the levels of plasma metabolites related to BCAA metabolism [20, 21, 28, 29, 60–62].

We also found xenometabolites were noticeably loaded in the metabolite pattern associated with physical activity. Xenobiotics are a group of chemicals not endogenously produced in organisms or in the environment. Humans are principally exposed to xenobiotics through dietary intake such as dietary supplements like antioxidants or drugs, antibiotics [63]. There are limited metabolomics studies exploring the associations of physical activity and circulating plasma xenobiotic metabolites [53, 64, 65], which hampered further interpretation of our findings.

Several studies have highlighted sex-differences in not only exercise habits and intensity [2, 38, 39, 66] but also within the phenotype of physiological systems and responses to exercise [67, 68]. Sex-differences in metabolite concentrations have also been described [40–42]. In the present study, we found no associations of physical activity with metabolome in females after correcting for multiple comparisons. We note that even though there is a dearth of evidence on leisure time physical activity and metabolome in young people, a recent longitudinal study found some alterations of serum amino acids in Finnish girls, specifically, isoleucine, leucine and tyrosine levels independent of BMI [29]. Their study differs from the present study in several ways. For example, the choice of serum vs. plasma, their methodology, and the potential confounders adjusted. Future metabolomics-based studies in this research area may consider these mixed findings and potential sources of variation. In light of growing evidence, other confounding variables such as hormonal contraceptive use should additionally be considered. For example, in a study exploring general variability of plasma metabolites in young adults, 97 metabolites differed significantly between females on hormonal contraceptives and those not on hormonal contraceptives [42].

Moreover, in our multivariable regression models, we found no associations of physical activity with single metabolites in plasma and urine in both sexes after corrections for multiple comparisons. Conscious of Altman and Bland’s “absence of evidence is not evidence of absence” [69], we offer a few considerations for interpretation of these non-significant findings. First, multiple-hypothesis-testing approach controlling for false discovery at 5% is demonstrably plausible at controlling type I errors for exploratory purposes, but may also be at a stringent level that increases the chances of type II errors, potentially rejecting biologically and statistically compelling associations. This is especially true when the effect sizes of the true positive hypotheses are small, or when the null hypothesis is true for a large number of tests, hence; true associations are missed due to the correction for multiple testing. The perils of our approach and future considerations are discussed in detail elsewhere [70, 71]. Nonetheless, to support future studies in this area, especially for integrative analysis purposes, we provided full list of metabolites, unadjusted and adjusted p-values, including metabolites with non-significant associations (Additional file **2**: Table **S1**).

Additionally, previous non-metabolomics studies have reported associations of acute exercise with alterations in levels of single metabolites, especially those involved in energy metabolism such as lactate [72–74]. Increased energy demand in muscles during physical training activates several metabolic pathways leading to increased production of lactate whose levels increase with exercise intensity [74]. In metabolomics-based studies, many of the metabolites whose global concentrations change significantly following exercise training are lipids and lipid-related metabolites such as free fatty acids and acylcarnitines [17–19, 75]. We considered that some metabolic pathways and enzymes that modulate levels of these metabolites such as lactate become activated by exercise and may be inactive at rest [75–77]. Alterations in such metabolite levels during and shortly after exercise training suggest acute physiological responses to physical exercise, perturbations that may not strongly persist when modelling habitual leisure time activities. In the present analysis, the implications of unmeasured variables such as the intensity and time lapse between last exercise activity and urine and blood collection are also unknown. However, by looking at habitual physical activity rather than a bout of exercise, we were more interested in the long-term effects of physical activity on metabolome because many health benefits of physical activity become apparent over extended timeframes, ranging from weeks to months, and even years as discussed here [12].

Choosing the ideal biological sample is critical in metabolomic profiling studies, but the most suitable choice for a specific research objective is an issue of scientific interest. For exercise physiology, blood (plasma or serum) is the most commonly used biosample in many studies as reported in this review [12]. However, there are inconsistencies in evidence as to whether the same metabolomic changes can be observed in different biological samples or tissues [12]. Although we observed statistically significant variation in a plasma metabolite pattern among males, it is not possible to definitively determine if the same metabolite variations or closely-related metabolite changes would have been observed in urine samples. This is because our analysis included overlapping samples to maximize the sample size, and only 136 participants had both urine and blood measurements and physical activity assessment. For a more robust comparison, the same set of participants should have both biosamples and physical measurements taken during the same period. We noted that metabolites with higher weights in our metabolite pattern were also reported in other exercise-metabolome studies in blood (plasma, serum) samples (Additional file **3**: Table **S2)**. Most of the studies included only male population and considered together with our results, it is still unclear if these findings reflect a sex-specific metabolite response to exercise or it is simply imbalanced research focus towards male subjects in most of exercise physiology studies.

Overall, our exploratory findings are quite intriguing and warrant further investigation in independent cohorts. The perturbations of the plasma metabolome in males are in molecular pathways of some health states and phenotypes. Non-esterified fatty acids, acylcarnitines, and phospholipids are suggested biomarkers for obesity as observed in many studies summarized by Rauschert et al. [78]. Circulating BCAA metabolites are widely recognized markers of body composition and obesity [23, 31, 79, 80] and proposed potential biomarkers of cardio metabolic health and diabetes [22–25]. Given the associations of physical activity and metabolic syndrome, further investigations may lend insights into some of the metabolic pathways through which habitual physical activity may influence metabolic health.

The present study has specific strengths that are worth highlighting. We used both untargeted metabolomics and untargeted lipidomics, which provided a comprehensive system-wide approach to explore and map molecular networks and perturbations in plasma and urine that may be associated with habitual physical activity. The study also explored two widely used biosamples — plasma and urine — and a relatively large sample size compared to other metabolomics studies. The sex-stratified investigations ensures that sex-specific relationships between metabolome and external stressors such as physical activity are not obfuscated. Our priori defined analyses investigating both single metabolites and metabolite patterns and correcting for type I errors, permitted a comprehensive exploration of the relationship between habitual physical activity urine and plasma metabolome, while also controlling for false positives associations.

There were nonetheless some notable limitations. Even though we used widely recognized and validated assessment instruments, dietary intake and physical activity data were self-reported; hence, subject to measurement errors such as an overestimation of physical activity as reported in other studies [81]. Multiple studies have reported a low to moderate correlation between objectively measured physical activity and self-reported measures in adolescents [82, 83]. The discrepancies between these measures may affect the associations observed in our study, but without objective measurements, the precise effects could not be determined. Lastly, the PCA approach is entirely unsupervised, and even though this may be a positive feature of the method depending on the research objective; it may also be a limitation as the relevant structures of the data do not consider variation with respect to the outcome or response variable [84]. Therefore, using factor weights to select top loading metabolites where PCA scores are associated with phenotype of interest or group information such as physical activity is data-driven, and its utility is descriptive and exploratory rather than inferential. Furthermore, 21 of the 82 top loading metabolites in PC15 are of unnamed biochemical compounds; hence, no meaningful biological information could be discerned.

## Conclusion

In summary, the current study suggests that habitual physical activity is associated with perturbations in the plasma metabolome. We observed that physical activity potentially influences the joint lipid, amino acid, and xenobiotics metabolism. Such metabolic alterations may reflect some of the important biological pathways that mediate the health effects of physical activity. However, these findings are exploratory and warrant further investigation in confirmatory studies.

## Electronic supplementary material

Below is the link to the electronic supplementary material.

**Additional file 1**: Metabolite data acquisition steps

**Additional file 2. Table S1**: Results of the multivariable regressions of single metabolites and metabolite pattern scores with physical activity in sex-stratified analysis

**Additional file 3. Figure S1**: Graphical presentation of metabolite contributions to PC15; **Figure S2**: Top loading metabolites in PC15; **Table S2**: Biochemical information of the top loading metabolites in PC15 (n = 82)

## Data Availability

The datasets generated and/or analyzed in the present study are subject to general data protection regulation; hence, not publicly available, but are available upon reasonable request from the corresponding author upon approval of the principal investigator. All results generated from the analyses are provided as supplementary files.
