# Supplementary material for: Exploring the association of physical activity with the plasma and urine metabolome in adolescents and young adults
Source: Nutr Metab (Lond). 2023 Apr 5;20:23. doi: 10.1186/s12986-023-00742-3 (PMC10074825; doi:10.1186/s12986-023-00742-3)
Supplement: Supplementary file 3 — Additional file 3. Figure S1: Graphical presentation of metabolite contributions to PC15; Figure S2: Top loading metabolites in PC15; Table S2: Biochemical information of the top loading metabolites in PC15 (n = 82) [file 12986_2023_742_MOESM3_ESM.docx]

**Supplementary material**

**Table of contents**

[Figure S1 Graphical presentation of metabolite contributions to PC15 2](#_Toc130291548)

[Selecting top metabolites 3](#_Toc130291549)

[Figure S2: Top loading metabolites in PC15 (n=82) 3](#_Toc130291550)

[Table S2: Biochemical information of the top loading metabolites in the metabolite pattern (PC15) associated with physical activity (n= 82). 4](#_Toc130291551)

[References 8](#_Toc130291552)

# Figure S1 Graphical presentation of metabolite contributions to PC15


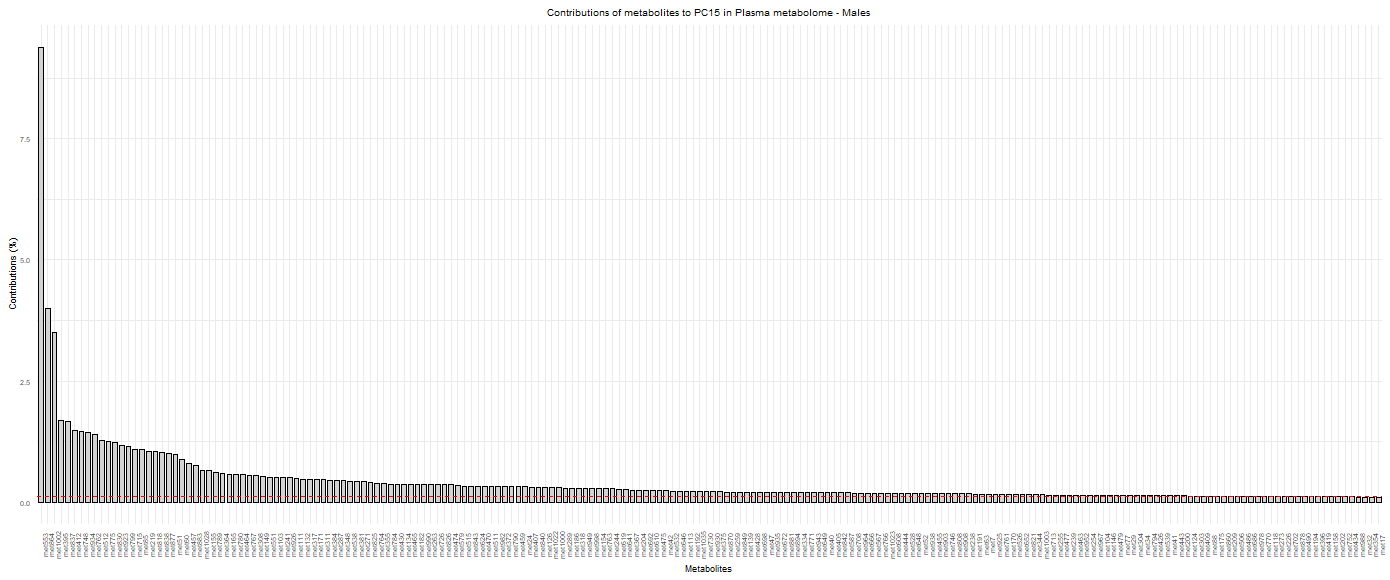


Figure S1: Graphical representation of contributions of metabolites in PC15. For practical reasons, only top n = 200 metabolites of n = 791 are shown here. The reference dashed line corresponds to the expected value if the contribution of all metabolites in this PC factor were uniform. Metabolites above this reference line can be considered as most important in contributing to the statistical information of PC15. Abbreviations are available in supplementary table S1.

# Selecting top metabolites

Since PCA employs a variance–covariance matrix for dimensionality reduction [1, 2], the absolute size of each metabolite was considered. Accordingly, this approach means that metabolites with larger values contributes more variance to the PC component than metabolites with smaller values. For males, we had *n* = 791 metabolites contributing to PC15, representing 100% (Figure S1). If all metabolites contributed equally, they would have a weight factor of approximately 0.13. Metabolites above this loading weight may be considered important variables to the metabolite pattern represented by PC15. Based on this, *n* = 161 metabolites were selected. To describe the most important of the selected metabolites, the median contribution weights were determined and considered those above this cut off (*n* = 82) as the most significant metabolites. This set of metabolites was also consistent with rotation matrix of PC15 (Figure S2). Even though our approach is descriptive [3], we considered it more informative compared to subjectively selecting some metabolites (e.g., top 10 or top 50 metabolites).

# Figure S2: Top loading metabolites in PC15 (n=82)


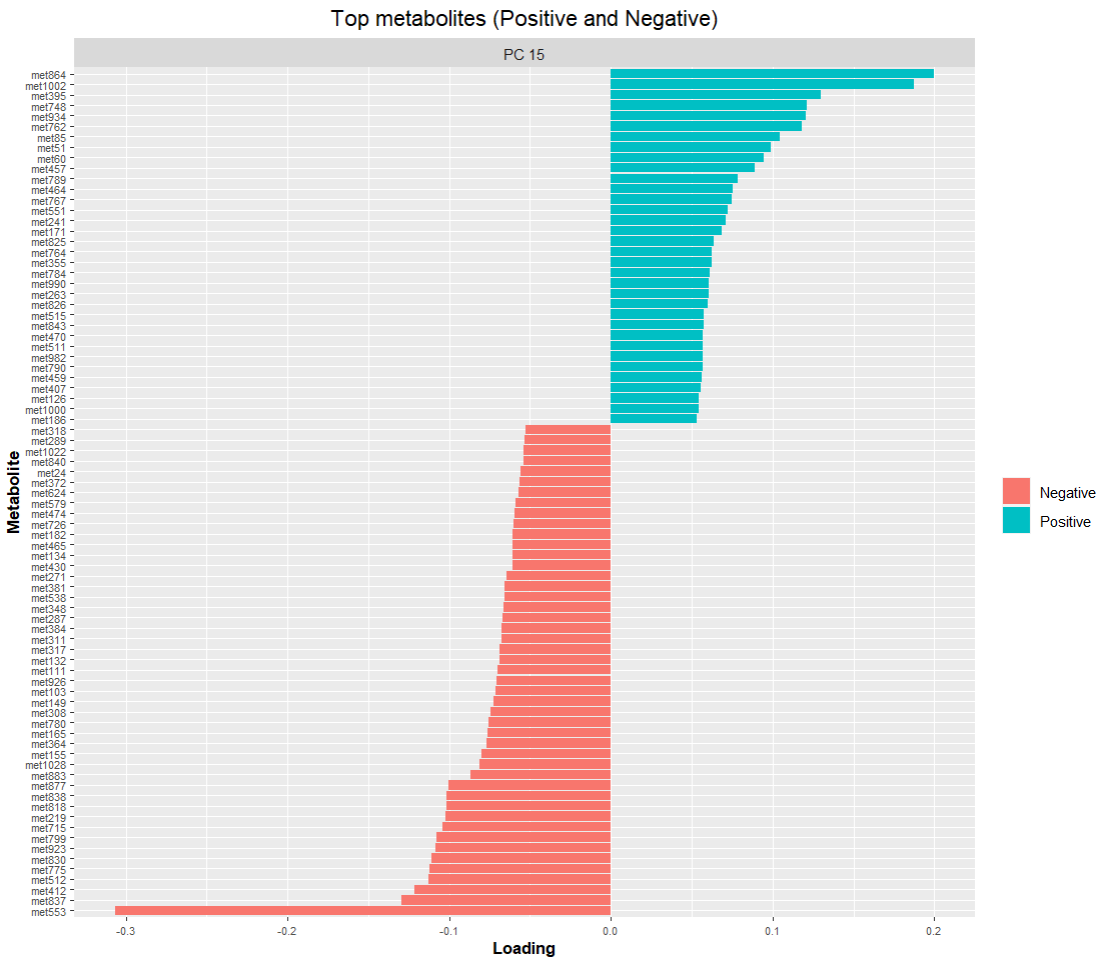


Figure S2: Top loading metabolites in PC15. Abbreviations are available in supplementary Table S1.

# Table S2: Biochemical information of the top loading metabolites in the metabolite pattern (PC15) associated with physical activity (n= 82).

| Code | HMDB ID | Biochemical compound | Super pathway | Sub pathway | Other studies in which the metabolite values varied with type of exercise and/or levels of physical activity | Biosample  Analyzed |
| --- | --- | --- | --- | --- | --- | --- |
| met24 | HMDB0240626 | 1-carboxyethylleucine | Amino Acid | Leucine, Isoleucine and Valine Metabolism | Resistance and endurance exercise training [4] | plasma |
| met51 |  | 21-hydroxypregnenolone monosulfate | Lipid | Pregnenolone Steroids | Resistance and endurance exercise training [4]  21-hydroxypregnenolone disulfate is associated with exercise endurance [4-6] | Plasma  Serum |
| met60 | HMDB00317 | 2-hydroxy-3-methylvalerate | Amino Acid | Leucine, Isoleucine and Valine Metabolism | Resistance [4] and aerobic exercise [7] | Plasma  Serum |
| met85 | HMDB00378 | 2-methylbutyrylcarnitine (C5) | Amino Acid | Leucine, Isoleucine and Valine Metabolism | Aerobic exercise [7], 3-day intensified exercise (vs pre-exercise)[8]. | Serum |
| met103 |  | 3-(methylthio)acetaminophen sulfate* | Xenobiotics | Drug - Analgesics, Anesthetics |  |  |
| met111 |  | 3beta-hydroxy-5-cholestecnoate | Lipid | Sterol |  |  |
| met126 | HMDB00413 | 3-hydroxydodecanedioate | Lipid | Fatty Acid, Dicarboxylate | Resistance and endurance exercise training [4] | Plasma |
| met132 |  | 3-hydroxymyristate | Lipid | Fatty Acid, Monohydroxy | Resistance and endurance exercise training [4] | Plasma |
| met134 |  | 3-hydroxyoleate | Lipid | Fatty Acid, Monohydroxy | Resistance exercise [4] | Plasma |
| met149 | HMDB00522 | 3-methylglutaconate | Amino Acid | Leucine, Isoleucine and Valine Metabolism | Resistance and endurance exercise training [4] | Plasma |
| met155 | HMDB00026 | 3-ureidopropionate | Nucleotide | Pyrimidine Metabolism, Uracil containing | Resistance and endurance exercise training [4] | Plasma |
| met165 | HMDB00921 | 4-cholesten-3-one | Lipid | Sterol | Significantly different between the resistance and endurance exercise groups (adjusted p <0.05), but not within each group separately. [4] | Plasma |
| met171 |  | 4-hydroxycoumarin | Xenobiotics | Drug - Cardiovascular | Resistance and endurance exercise training [4] | Plasma |
| met182 |  | 4-vinylguaiacol sulfate | Xenobiotics | Food Component/Plant |  |  |
| met186 | HMDB00076 | 5,6-dihydrouracil | Nucleotide | Pyrimidine Metabolism, Uracil containing | Significantly different between the resistance and endurance exercise groups (adjusted p <0.05), but not within each group separately. [4] | Plasma |
| met219 | HMDB04704 | 9,10-DiHOME | Lipid | Fatty Acid, Dihydroxy | Resistance and endurance exercise training [4] | Plasma |
| met241 | HMDB02759 | andro steroid monosulfate C19H28O6S (1)* | Lipid | Androgenic Steroids | Resistance and endurance exercise training [4] | Plasma |
| met263 | HMDB00191 | aspartate | Amino Acid | Alanine and Aspartate Metabolism | Acute exercise [9] | Plasma |
| met271 | HMDB00043 | betaine | Amino Acid | Glycine, Serine and Threonine Metabolism | Resistance and endurance exercise training [4], acute exercise [9], physical activity in community dwelling population [10]. | Plasma |
| met287 | HMDB00808 | butyrylglycine (C4) | Lipid | Fatty Acid Metabolism (also BCAA Metabolism) |  |  |
| met289 | HMDB01847 | caffeine | Xenobiotics | Xanthine Metabolism | Resistance and endurance exercise training [4] | Plasma |
| met308 | HMDB00619 | cholate | Lipid | Primary Bile Acid Metabolism | 3-day intensified exercise (vs pre-exercise)[8], acute exercise [9], and resistance and endurance exercise [4] | Serum, plasma |
| met311 | HMDB00097 | choline | Lipid | Phospholipid Metabolism | Resistance and endurance exercise training [4], Acute exercise [9], endurance exercise [11] | Plasma  Blood |
| met317 | HMDB60463 | citalopram propionate* | Xenobiotics | Drug – Psychoactive |  |  |
| met318 | HMDB05028 | citalopram/escitalopram | Xenobiotics | Drug – Psychoactive |  |  |
| met348 |  | desmethylnaproxen sulfate | Xenobiotics | Drug - Analgesics, Anesthetics |  |  |
| met355 |  | dihydroferulate | Xenobiotics | Food Component/Plant | Resistance and endurance exercise training [4] | Plasma |
| met364 |  | docosahexaenoylcholine | Lipid | Fatty Acid Metabolism (Acyl Choline) | Resistance and endurance exercise training [4] | Plasma |
| met372 | HMDB06275 | dopamine 3-O-sulfate | Amino Acid | Tyrosine Metabolism | Resistance and endurance exercise training [4] | Plasma |
| met381 |  | eicosenedioate (C20:1-DC)* | Lipid | Fatty Acid, Dicarboxylate |  |  |
| met384 |  | epiandrosterone sulfate | Lipid | Androgenic Steroids | Resistance and endurance exercise training [4] | Plasma |
| met395 |  | eugenol sulfate | Xenobiotics | Food Component/Plant | Resistance and endurance exercise training [4] | Plasma |
| met407 |  | gamma-CEHC glucuronide* | Cofactors and Vitamins | Tocopherol Metabolism |  |  |
| met412 | HMDB03869 | gamma-glutamyl-epsilon-lysine | Peptide | Gamma-glutamyl Amino Acid | Resistance exercise training [4] | Plasma |
| met430 | HMDB00122 | glucose | Carbohydrate | Glycolysis, Gluconeogenesis, and Pyruvate Metabolism | Resistance exercise training [4] , Acute exercise [9] | Plasma |
| met457 |  | glycocholenate sulfate | Lipid | Secondary Bile Acid Metabolism | Resistance and endurance exercise training [4] | Plasma |
| met459 |  | glycodeoxycholate 3-sulfate | Lipid | Secondary Bile Acid Metabolism | Significantly different between the resistance and endurance exercise groups (adjusted p <0.05), but not within each group separately. [4] | Plasma |
| met464 | HMDB60013 | guaiacol sulfate | Xenobiotics | Benzoate Metabolism |  |  |
| met465 | HMDB00128 | guanidinoacetate | Amino Acid | Creatine Metabolism | Exercise regulated in healthy males [4] | Plasma |
| met470 |  | heptenedioate (C7:1-DC) | Lipid | Fatty Acid, Dicarboxylate | Resistance exercise training [4] | Plasma |
| met474 | HMDB00705 | hexanoylcarnitine (C6) | Lipid | Fatty Acid Metabolism (Acyl Carnitine, Medium Chain) | Resistance exercise training [4] | Plasma |
| met511 | HMDB00718 | isovalerate (C5) | Amino Acid | Leucine, Isoleucine and Valine Metabolism | Resistance exercise training [4], acute exercise [9]. | Plasma |
| met512 | HMDB00688 | isovalerylcarnitine (C5) | Amino Acid | Leucine, Isoleucine and Valine Metabolism | Resistance exercise training [4], aerobic exercise [7], 3-day intensified exercise (vs pre-exercise)[8] | Plasma  Serum |
| met515 | HMDB00684 | kynurenine | Amino Acid | Tryptophan Metabolism | Resistance and endurance exercise training [4], Acute exercise [9], aerobic exercise marker [12] | Plasma  Serum |
| met538 | HMDB00169 | mannose | Carbohydrate | Fructose, Mannose and Galactose Metabolism | Resistance and endurance exercise training [4] | Plasma |
| met551 | HMDB00202 | methylmalonate (MMA) | Lipid | Fatty Acid Metabolism (also BCAA Metabolism) | Resistance and endurance exercise training [4] | Plasma |
| met553 | HMDB01844 | methylsuccinate | Amino Acid | Leucine, Isoleucine and Valine Metabolism | Resistance and endurance exercise training [4] | Plasma |
| met579 | HMDB01325 | N6,N6,N6-trimethyllysine | Amino Acid | Lysine Metabolism | Resistance and endurance exercise training [4], Acute exercise [9] | Plasma |
| met624 |  | N-oleoylserine | Lipid | Endocannabinoid |  |  |
| met715 | HMDB01358 | retinal | Cofactors and Vitamins | Vitamin A Metabolism | Acute exercise [9], resistance and endurance exercise training [4] | Plasma |
| met726 | HMDB00271 | sarcosine | Amino Acid | Glycine, Serine and Threonine Metabolism | Aerobic exercise training in males [12] | Serum |
| met748 | HMDB31554 | sucralose | Xenobiotics | Food Component/Plant |  |  |
| met762 | HMDB00896 | taurodeoxycholate | Lipid | Secondary Bile Acid Metabolism | 3-day intensified exercise (vs pre-exercise)[8], acute exercise [9] | Plasma |
| met764 | HMDB02580 | taurolithocholate 3-sulfate | Lipid | Secondary Bile Acid Metabolism | Resistance exercise training [4], 3-day intensified exercise (vs pre-exercise)[8]. |  |
| met767 | HMDB00560 | tetradecadienoate (14:2) | Lipid | Long Chain Polyunsaturated Fatty Acid (n3 and n6) | Resistance and endurance exercise training [4],  3-day intensified exercise (vs pre-exercise)[8] | Plasma  Serum |
| met775 | HMDB00943 | threonate | Cofactors and Vitamins | Ascorbate and Aldarate Metabolism | Resistance and endurance exercise training [4], Acute exercise [9] | Plasma |
| met780 | HMDB00725 | hydroxyproline | Amino Acid | Urea cycle; Arginine and Proline Metabolism | Acute exercise [9] | Plasma |
| met784 | HMDB00925 | trimethylamine N-oxide (TMAO) | Lipid | Phospholipid Metabolism | Resistance and endurance exercise training [4], Acute exercise [9], moderate to vigorous physical activity [13], 12 weeks exercise training [14] | Plasma |
| met789 |  | umbelliferone sulfate | Xenobiotics | Food Component/Plant |  |  |
| met790 | HMDB00888 | undecanedioate (C11-DC) | Lipid | Fatty Acid, Dicarboxylate |  |  |
| met799 | HMDB01877 | valproate (2-propylpentanoate) | Xenobiotics | Drug – Neurological |  |  |
| met818 |  | X – 11372 |  |  |  |  |
| met825 |  | X – 11470 |  |  |  |  |
| met826 |  | X - 11478 |  |  |  |  |
| met830 |  | X - 11632 |  |  |  |  |
| met837 |  | X - 11852 |  |  |  |  |
| met838 |  | X - 11858 |  |  |  |  |
| met840 |  | X - 12007 |  |  |  |  |
| met843 |  | X - 12026 |  |  |  |  |
| met864 |  | X - 12729 |  |  |  |  |
| met877 |  | X - 13431 |  |  |  |  |
| met883 |  | X - 13866 |  |  |  |  |
| met923 |  | X - 18899 |  |  |  |  |
| met926 |  | X - 18921 |  |  |  |  |
| met934 |  | X - 21310 |  |  |  |  |
| met949 |  | X - 21471 |  |  |  |  |
| met982 |  | X - 23739 |  |  |  |  |
| met990 |  | X - 24295 |  |  |  |  |
| met1000 |  | X - 24455 |  |  |  |  |
| met1002 |  | X - 24475 |  |  |  |  |
| met1022 |  | X - 25217 |  |  |  |  |
| met1028 |  | X – 25371 |  |  |  |  |

# References

1. Jolliffe, I.T. and J. Cadima, *Principal component analysis: a review and recent developments.* Philos Trans A Math Phys Eng Sci, 2016. **374**(2065): p. 20150202.

2. Kitao, A., *Principal Component Analysis and Related Methods for Investigating the Dynamics of Biological Macromolecules.* J, 2022. **5**(2): p. 298-317.

3. Yamamoto, H., et al., *Statistical hypothesis testing of factor loading in principal component analysis and its application to metabolite set enrichment analysis.* BMC Bioinformatics, 2014. **15**(1): p. 51.

4. Morville, T., et al., *Plasma Metabolome Profiling of Resistance Exercise and Endurance Exercise in Humans.* (2211-1247 (Electronic)).

5. Al-Khelaifi, F., et al., *A pilot study comparing the metabolic profiles of elite-level athletes from different sporting disciplines.* (2199-1170 (Print)).

6. Tarkhan, A.H., et al., *Comparing metabolic profiles between female endurance athletes and non-athletes reveals differences in androgen and corticosteroid levels.* The Journal of Steroid Biochemistry and Molecular Biology, 2022. **219**: p. 106081.

7. Margolis, L.M., et al., *Serum Branched-Chain Amino Acid Metabolites Increase in Males When Aerobic Exercise Is Initiated with Low Muscle Glycogen.* Metabolites, 2021. **11**(12).

8. Nieman, D.C., et al., *Serum metabolic signatures induced by a three-day intensified exercise period persist after 14 h of recovery in runners.* J Proteome Res, 2013. **12**(10): p. 4577-84.

9. Nayor, M., et al., *Metabolic Architecture of Acute Exercise Response in Middle-Aged Adults in the Community.* (1524-4539 (Electronic)).

10. Fukai, K.A.-O., et al., *Metabolic Profiling of Total Physical Activity and Sedentary Behavior in Community-Dwelling Men.* (1932-6203 (Electronic)).

11. Penry, J.T. and M.M. Manore, *Choline: an important micronutrient for maximal endurance-exercise performance?* (1526-484X (Print)).

12. Felder, T.K., et al., *Specific circulating phospholipids, acylcarnitines, amino acids and biogenic amines are aerobic exercise markers.* (1878-1861 (Electronic)).

13. Argyridou, S.A.-O., et al., *Associations between physical activity and trimethylamine N-oxide in those at risk of type 2 diabetes. LID - 10.1136/bmjdrc-2020-001359 [doi] LID - e001359.* (2052-4897 (Electronic)).

14. Erickson, M.L., et al., *Effects of Lifestyle Intervention on Plasma Trimethylamine N-Oxide in Obese Adults. LID - 10.3390/nu11010179 [doi] LID - 179.* (2072-6643 (Electronic)).
